# Impact on quality of life of keratoconus patients treated with accelerated “epi-on” corneal collagen crosslinking technique: results from the NEI VFQ-25 Questionnaire in a Romanian population

**DOI:** 10.22336/rjo.2023.48

**Published:** 2023

**Authors:** Alina-Cristina Chiraples, Horia Stanca, Daiana Mărgărit, Mihnea Munteanu

**Affiliations:** * Department of Ophthalmology, “Victor Babeş” University of Medicine and Pharmacy, Timişoara, Romania; ** Department of Ophthalmology, “Carol Davila” University of Medicine and Pharmacy, Bucharest, Romania

**Keywords:** keratoconus, accelerated “epi-on” corneal collagen crosslinking, NEI VFQ-25, demographics

## Abstract

**Objective:** The main objective of this study was to describe vision-related quality of life (VRQoL) in a Romanian population of patients with keratoconus who underwent the accelerated “epi-on” corneal collagen crosslinking procedure and to evaluate the association with demographic data of age, gender and civil status (married, cohabitants or non-cohabitants).

**Method:** The National Eye Institute Visual Functioning Questionnaire-25 (NEI VFQ-25) was administered to 72 patients with keratoconus who had undergone a minimally invasive procedure. Descriptive statistics and bivariate analysis were used to determine the distribution of demographic parameters and a possible association between this parameter and the scores obtained on the NEI VFQ-25 questionnaire.

**Results:** Based on the answers to the questionnaire we calculated the mean (SD) VFQ-25 total score, which was 73,97 (15,11), whilst scores for the subscales varied from 49.93 to 84,23. No significant difference was observed between the demographic parameters and the NEI VFQ-25 items, except for one item (being with others) when comparing paired and non-paired participants.

**Conclusion:** In a Romanian population with keratoconus being treated with accelerated “epi-on” corneal collagen crosslinking procedure, VRQoL was reported at a high baseline level. The value of this information is significant when discussing patients’ expectations during treatment. The VRQoL was not affected by age or civil status.

## Introduction

Defined for the first time in 1854, keratoconus is a chronic disease. Keratoconus is a bilateral non-inflammatory progressive corneal thinning and bulging that is usually diagnosed in adolescence [**1**]. Less commonly, this ectatic corneal disease can develop in children and is more frequently associated with rapid progress compared with adults [**1**]. Ectasia is defined as permanent or temporary swelling of a tubular or cavitary organ. Although is considered the most common corneal ectasia, keratoconus has a small frequency, with an incidence of 2 cases at 100000 people and a prevalence of 54,5 at 100000 people [**2**]. A geographic variation is present. The disease is bilateral and asymmetrical, at its starting point affecting only one eye. Approximately 50% of the patients without a binocular form will develop symptoms in the second eye in the next 16 years [**3**]. At the starting point, keratoconus can have minimum symptoms, whereas in advanced forms there can be profound visual loss [**2**].

Keratoconus is associated with corneal steepening, visual distortion, apical corneal thinning, and central corneal scarring. Astigmatism and myopia occur because of corneal thinning, leading to mild or marked visual impairment [**2**].

Various means of treatment have been utilized over time, and nowadays the minimum invasive techniques, like accelerated “epi-on” corneal collagen crosslinking, are preferred. This method has numerous advantages including rapidity, which increases patient compliance, fast healing, long long-lasting effects (compared with hard contact lenses).

 It is important to correlate the treatment effects, epidemiology, genetics, risk factors, and quality of life (QoL) to better understand how people live with Keratoconus. Aspects like psychological well-being, daily functioning, emotional status, and social activity cannot be captured by one single clinical measurement. 

According to previous psychometric studies, the results obtained by submitting the NEI VFQ-25 Questionnaire are comparable with the ones of the survey that consist of 51 items [**4**]. Therefore, the sub-scores of NEI VFQ-25 correlate different answers for a better understanding of the impact that the disease has on a daily schedule. NEI VFQ-25 has been used in many studies examining different eye diseases like age-related macular degeneration (AMD), glaucomatous field loss, cataract, Behcet uveitis, after penetrating keratoplasty for keratoconus, after retinal detachment surgery, vitrectomy, diabetic retinopathy, strabismus, multiple sclerosis and low vision of any cause [**4**]. The National Eye Institute Visual Functioning Questionnaire-25 (NEI VFQ-25) is a disease-specific instrument that measures VRQoL. In the literature, many studies, such as Chinese (Yank K et al., 2021), Japanese (Tatematsu-Ogawa Y et al., 2008) and Turkish (Kurna et al., 2014) exist, which have used NEI VFQ-25 to determine the visual related QoL. To date, no Romanian studies with this purpose have been performed. 

This study aimed to describe VRQoL measured by NEI VFQ-25 in a Romanian population diagnosed with keratoconus and treated using the accelerated “epi-on” corneal collagen crosslinking method. This research also aimed to evaluate the association with demographic data like gender, age, and civil status.

## Method

*Participants and Study Design*

This cross-sectional study comprises patients with clinical diagnoses of keratoconus, who have undergone an accelerated “epi-on” corneal crosslinking procedure. Participants were recruited at Prof. Munteanu Ophthalmologic Center, Timişoara, between October 2019 and August 2022. The participants were adults, of both genders, with different civil statuses (married, cohabitants, and non-cohabitants), without any documented cognitive impairment. All of them have completed a written consent form before attending the study.

*Data Collection Procedures and Measures*

This study included baseline data from 72 patients treated with accelerated “epi-on” corneal collagen crosslinking for keratoconus.

Due to the pandemic situation, to minimize the time spent in the clinic, the data collection was submitted by interviewer-administered questionnaire by telephone.

The NEI VFQ-25 Questionnaire

The National Eye Institute from Maryland, USA, has developed this questionnaire, as a shorter version of the 51-item NEI VFQ. From the total of 25 questions, 11 sub-scales were generated for the following dimensions of vision-target quality of life (QoL): general vision, difficulty with near-vision activities, difficulty with distance-vision activities, limitation in social functioning, role limitation, dependency on others, vision-related mental health symptoms, driving difficulties, limitations in peripheral vision, alterations in color vision, ocular pain. Additionally, there was a single-item general health rating question. When obtaining the score for NEI VFQ-25, one had to transform the pre-coded numeric values to a score from 0 to 100 using the VFQ-25 manual. A 0 score refers to the worst possible score and a 100 score describes the best possible score. It is possible to calculate an overall composite score for the VFQ-25 Questionnaire by simply averaging the vision-targeted sub-scale scores.

**Table 1:** Demographic Data referring to the study subjects. Total patient sample (n = 72) %

	Total patient sample (n=72)	%
Female/male	20/52	27,7/72,2
Age, years		100
M(SD)	35,1(7,9)	
Range	20-56	
Age 20-30	13	18
Age 30-40	42	58,5
Age 40-50	13	18
Age>50	4	5,5
Paired/Non-paired	49/13	68/32

*Statistical Methods*

Microsoft Office Excel, version 2003 was used to analyze the data. The appliance of descriptive statistics was useful in determining the distribution of demographic and clinical characteristics. Referring to the vision-related quality of life, the primary outcome was measured with the NEI VFQ-25 categories such as work-related duties, seeing in brightness, being capable of watching movies and sports, general trouble at seeing, diffuse illumination, driving at night, driving in the day time, emotional mood, mental health, seeing clearly, reading.

When describing the population, the following characteristics were considered: age, gender, and marital status. Bivariate analyses were performed to determine the association between demographic factors and NEI VFQ-25 scores. The population was divided into decades of age between 20 and 50s and the ones over 50 years formed a separate category. According to their civil status, the participants were divided into paired (including married and cohabitants) and non-paired (including single patients).

## Results

*Descriptive Analyses - The demographics and characteristics of the population*

72 patients completed the questionnaire. Whilst their mean age was 32,16 years, the standard deviation (SD) was 7,92, having a range from 20 years old to 56. The group consisted of 27,7% females and 72,2% males and 68% of the population was paired and 32% non-paired. A more detailed description of the attendees is shown in **Table 1**.

*NEI VFQ-25 Scores*

The overall composite score had a mean of 73,97. Referring to the sub-scores, a mean that varied from 49,93 corresponding to the “Ocular pain” subscale until 81,94 resulted from the “Color vision” subscale. “General vision” and “Ocular pain” revealed the lowest score. Separately from the vision-related subscales, there was the “General health” sub-scale with a mean equal to 84,23. **Table 2** shows a more detailed description of the sub-scores.

**Table 2:** Each Subscale scores and the Total Score of NEI VFQ-25

Subcale NEI VFQ-25 questionnaire	M	SD	Range	n
General health	84,23	15,55	50-100	72
General vision	64,2	13,34	32,25-100	72
Ocular pain	49,93	13,66	17,5-50	72
Near activities	76,03	18,17	25-100	72
Distance activities	71,94	21,62	25-100	72
Social functioning	71,87	26,93	0-100	72
Mental health	72,15	13,7	45-100	72
Role difficulties	79,86	12,06	50-100	72
Dependency	79,34	13,04	31,25-100	72
Driving	76,73	21,54	0-100	65
Color vision	81,94	14,66	50-100	72
Peripheral vision	80,2	13,22	50-100	72
Total score	73,97	15,11	34,20-97,91	72
NEI VFQ-25 = National Eye Institute Visual Functioning Questionnaire				

*NEI VFQ-25 Age-related scores*

**Fig. 1:**
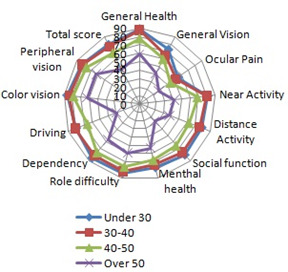
NEI VFQ-25 age-related subscales scores

Comparing the mean composite score for the 4 age categories, that the studies had, led to the largest mean found in the group under 30 years and its value was 77.9, and the lowest was found in the group between 30 and 40 years. For all 4 groups of age, the “Ocular pain” sub-scale had the lowest means, and the highest values were found in the “General Health” sub-scale, except the “Over 50 years” category. This could be explained by the common comorbidities that could appear with age (arterial high pressure, hypercholesterolemia, etc.). **Fig. 1** presents the comparison between the mean scores of the age groups and the NEI VFQ-25 items. 

*NEI VFQ-25 Gender-related scores*

Mean composite scores by gender were 78.31 for women and 72,25 for men. The scores per subscales varied from 54,34 referring to Ocular pain to 88,15 corresponding to General health, among the female participants, and from 48,17 to 83,54 for men, concerning the same sub-scores.

**Fig. 2:**
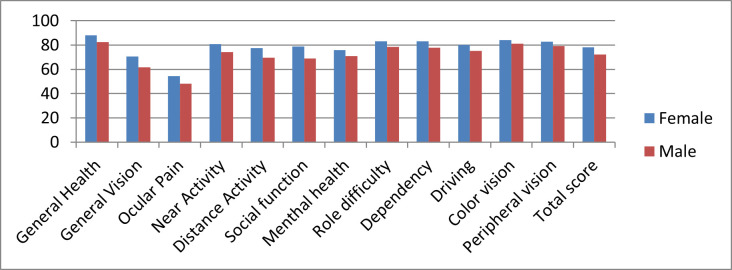
NEI VFQ-25 gender-related subscales scores

The highest difference between the 2 genders was calculated in the “Social functioning” sub-scale, but it did not have statistical significance. **Fig. 2** shows the mean scores by gender.

*NEI VFQ-25 Civil status-related scores*

The mean of total scores for the NEI VFQ-25 were 74,87 for paired patients and 72,07 for non-paired ones. The sub-scale values were the lowest in the “Ocular pain” category with a mean of 50,62 involving paired patients and 48,54 referring to the non-paired ones. The highest numbers were generated in the sub-score involving “General health”. The mean for paired attendees was 84,58, while for single patients was 83,54.

**Fig. 3:**
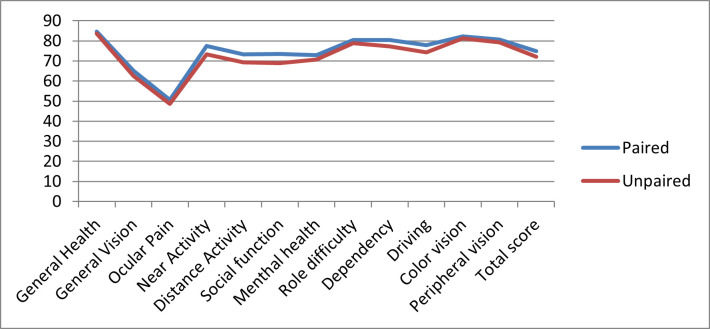
NEI VFQ-25 Civil status-related subscales scores

The mean sub-scores for other categories can be observed in **Fig. 3**. 

*Correlation Analyses*

The correlation matrix found in **Table 3** defined the link between the 12 subscales of the NEI VFQ-25 Questionnaire.

*International Comparison*

**Fig. 4:**
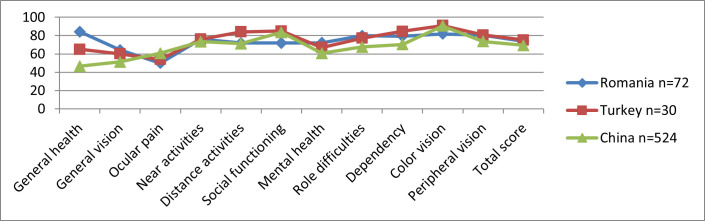
NEI VFQ-25 subscales score in different studies with Keratoconus patients

A comparison between studies using the same NEI VFQ-25 Questionnaire was made in different countries like China (Yang K et al., 2021) and Turkey (Kurna SA et al., 2014). As **Fig. 4** shows, there were some sub-scores with similar values for all 3 studies, like Ocular pain and Mental health, while the other sub-scores differed. This could be explained by the differences regarding the health system, geographic distance, and population mentality. 

**Table 3:** Correlation Matrix

	GH	GV	OP	NV	DV	SF	MH	RD	De	Dr	CV	PV
GH	1											
GV	0,6867	1										
OP	0,9137	0,8974	1									
NV	0,9231	0,9097	0,9842	1								
DV	0,8505	0,9471	0,9569	0,9768	1							
SF	0,9231	0,9097	0,9842	1	0,9768	1						
MH	0,5017	0,9232	0,7955	0,7681	0,8276	0,7681	1					
RD	0,9273	0,8787	0,9859	0,9811	0,9737	0,9811	0,7622	1				
De	0,7572	0,9739	0,902	0,9368	0,9873	0,9386	0,9811	0,7622	1			
Dr	0,9228	0,8680	0,9460	0,9820	0,9589	0,9820	0,7296	0,9570	0,9137	1		
CV	0,5451	0,7259	0,6772	0,6827	0,7669	0,6827	0,7383	0,7254	0,7875	0,6996	1	
PV	0,4755	0,7610	0,6893	0,6643	0,7276	0,6643	0,8980	0,6882	0,7662	0,6891	0,7905	1
GH = General Health, GV = General Vision, OP = Ocular Pain, NV = Near Vision, DV = Distance Vision, SF = Social Functioning, MH = Mental Health, RD = Role Difficulties, De = Dependency, Dr = Driving, CV = Colour Vision, PV = Peripheral Vision												

## Discussion

This study was conducted on a Romanian population with keratoconus treated with accelerated “epi-on” corneal collagen crosslinking, and it showed that VRQoL had high values. Results of the subscale referring to General health had the highest values (M = 84,23, SD = 15,55). The age range of the population (20-56) could explain that, with a mean of 35,1, keratoconus being diagnosed at an early age.

The vision-related sub-scales “Color vision” and “Peripheral vision” also had high values. The data is comparable with the one obtained in other studies [**5**,**6**]. These two categories present less alteration in keratoconus than in other ocular pathologies (like Glaucoma [**7**] and Retinitis Pigmentosa [**8**]). It showed that most of the study participants did not have difficulties picking and matching their clothes because their color vision was almost not altered and they also noticed objects off to the side whilst walking because the peripheral vision was low impaired. 

Low scores for sub-scales referring to “Ocular pain” and “General vision” were registered. The mean for “Ocular pain” was the lowest (49,93) and had an SD of 13,66. A percent of 6,94 of the attendees reported severe ocular pain like burning, itching, or aching and 33% considered their ocular pain to be moderate. Being questioned about how much the pain or discomfort in or around their eyes kept them from their activity, 4,16% of the participants answered that all the time and 18,05% most of the time. Regarding the “General vision” sub-scale, a mean equal to 64,2 was calculated, SD being 13,34. From the total, 8,3% of patients considered their vision to be poor and 4,16% marked their eyesight with 4 out of 10. Although the percentage could seem low, their significance could be revealed in correlation with the demographic data (the sample population was made up of young, active adults). The scores obtained in “Ocular pain” and “General vision” resembled the ones described by other studies that debated the quality of life in Keratoconus patients [**5**,**9**]. 

The “Distance activities” sub-scale also has low scores, with a mean equal to 71,94 and an SD of 21,62, representing poor vision at distance. 5,55% of the participants confirmed that they could express their difficulty in reading street signs, they could not read store names and it was a burden for them to go down on the stairs in dim light or at night. 

The “Near activities” sub-scale had a mean of 76,06, the SD being equal to 18,17. 20,83% of the participants in this study replied that they encountered moderate or extreme difficulties reading ordinary print in newspapers or seeing well up close, such as cooking, sewing, fixing things around the house, or using hand tools. 

A close connection could be considered between the “Mental health” score and the “Social functioning” score, with similar values. The conclusion that resulted was that they influenced each other. The emotional mood of the patients, the irritability, and the lack of confidence given by their ocular disease could have influenced both their mental state and their social life. Regarding the “Mental health” sub-scale, the results were comparable with other research met in the studies of the VR-QoL in a Turkish [**5**] or Chinese [**6**] population. A study about the impact of low vision on well-being showed that the patients’ psychological symptoms including tiredness, irritability, and absence of interest in daily activities might have been accompanied by negative feelings like sadness, anxiety, mood lability, and tearfulness going all the way to depression [**10**]. 

The “Dependency” sub-scale had a score of 79,34, with most of the patients declaring that most of the time they did not rely on others, they did not need help from others nor did they worry about doing embarrassing things. Referring to the “Driving” sub-scale, the results might be misleading. 9,72% of the participants did not drive, 6,94% because of other reasons, and 2,77% because of their eyesight. The other reasons for giving up driving could include financial causes, but also psycho-emotional reasons (which were not sufficiently discussed). Driving could be highly associated with independence, especially in countries with a limited or unavailable public transport system, due to that the value of the mean in the “Driving” sub-scale (76,73) could be correlated with the mean value of the “Dependency” sub-scale (79,34). The composite score had a mean equal to 73,97, similar to the studies conducted in Turkey [**5**] (M=75,2) and China [**6**] (M=69,35).

“Ocular pain” and “General vision” might have had the highest values due to the high expectations generated by the young age of the targeted participants. Compared with the elderly, most of the patients included in the study did not experience other ocular diseases like for example presbyopia and Sicca syndrome (frequently associated with old age).

**Strengths and Limitations**

The most important strength is the use of a standardized instrument like the NEI VFQ-25 Questionnaire. Thus, the possibility of comparing data with other available studies concerning the same disease. Although it is necessary to extend the research for identifying the causal factors for the alteration of the VRQoL, the submitted data has significant importance for explaining the affected visual-related QoL in the Romanian social, economic, and cultural context. 

When discussing the limitations, it is necessary to mention the lack of data from a reference group of healthy participants. This can be explained by the small number of screening consults, and the Romanian population having low medical addressability. Another limitation is that the NEI VFQ-25 questionnaire was created and adapted to the American language and culture, therefore some linguistic gaps might exist.

## Conclusions

Visual Related QoL was reported at a high baseline level, in a Romanian population with keratoconus being treated with accelerated “epi-on” corneal collagen crosslinking procedure. The value of this information is significant when discussing patients’ expectations during treatment. The VRQoL was not affected by age or civil status. Different sub-scales of the questionnaire show different results. The lowest values refer to ocular pain, with the majority of the subjects declaring that have suffered itching, aching, or pain in the eye. Another low value is registered in the sub-scale of general vision because the patients affected by this disease are young adults with active lifestyles. But, all in all, the dependency, social functioning, and other parameters have scored medium values, identifying keratoconus not being a debilitating disease.

**Conflict of Interest statement**

The author(s) declared no potential conflicts of interest concerning the research, authorship, and/or publication of this article.

**Informed Consent and Human and Animal Rights statement**

Informed consent has been obtained from all individuals included in this study.

**Authorization for the use of human subjects**

Ethical approval: The research related to human use complies with all the relevant national regulations and institutional policies, is by the tenets of the Helsinki Declaration, and has been approved by the Ethical Committee of “Victor Babeş” University of Medicine and Pharmacy, Timişoara, Timiş County.

**Acknowledgments**

None.

**Sources of Funding**

The author(s) received no financial support for the research, authorship, and/or publication of this article. 

**Disclosures**

None.

## References

[1] Al Suhaibani AH, Al-Rajhi AA, Al-Motowa S, Wagoner MD (2007). Inverse relationship between age and severity and sequelae of acute corneal hydrops associated with keratoconus. Br J Ophthalmol.

[2] Duncan JK, Esquenazi I, Weikert MP (2017). New Diagnostics in Corneal Ectatic Disease. International Ophthalmology Clinics.

[3] Bowling B, Kanski JJ (2016). Kanski’s clinical ophthalmology: a systematic approach.

[4] Mangione CM, Lee PP, Gutierrez PR, Spritzer K, Berry S, Hays RD (2001). National Eye Institute Visual Function Questionnaire Field Test Investigators. Development of the 25-item National Eye Institute Visual Function Questionnaire. Arch Ophthalmol.

[5] Aydin Kurna S, Altun A, Gencaga T, Akkaya S, Sengor T (2014). Vision related quality of life in patients with keratoconus. J Ophthalmol.

[6] Yang K, Xu L, Fan Q (2021). A hospital-based study on clinical data, demographic data and visual function of keratoconus patients in Central China. Sci Rep.

[7] Nassiri N, Mehravaran S, Nouri-Mahdavi K, Coleman AL (2013). National Eye Institute Visual Function Questionnaire: usefulness in glaucoma. Optom Vis Sci.

[8] Azizi F, Aghazadeh Amiri M, Riazi A, Norouzzadeh H, Tabatabaee S (2018). Quality of Life in Patients with Retinitis Pigmentosa in Shiraz. Scientific Journal of Rehabilitation Medicine.

[9] Mahdaviazad H, Bamdad S, Roustaei N, Mohaghegh S (2018). Vision-Related Quality of Life in Iranian Patients With Keratoconus: National Eye Institute Vision Function Questionnaire-25. Eye Contact Lens.

[10] Mojon-Azzi SM, Sousa-Poza A, Mojon DS (2008). Impact of low vision on well-being in 10 European countries. Ophthalmologica.

